# Comparison of the diagnostic performance of microscopic examination with nested polymerase chain reaction for optimum malaria diagnosis in Upper Myanmar

**DOI:** 10.1186/s12936-017-1765-4

**Published:** 2017-03-16

**Authors:** Jung-Mi Kang, Pyo-Yun Cho, Mya Moe, Jinyoung Lee, Hojong Jun, Hyeong-Woo Lee, Seong Kyu Ahn, Tae Im Kim, Jhang Ho Pak, Moe Kyaw Myint, Khin Lin, Tong-Soo Kim, Byoung-Kuk Na

**Affiliations:** 1 Department of Parasitology and Tropical Medicine, and Institute of Health Sciences, Gyeongsang National University School of Medicine, Jinju, 52727 Republic of Korea; 2 BK21Plus Team for Anti-Aging Biotechnology and Industry, Department of Convergence Medical Science, Gyeongsang National University, Jinju, 52727 Republic of Korea; 3 Department of Tropical Medicine and Inha Research Institute for Medical Sciences, Inha University School of Medicine, Incheon, 22212 Republic of Korea; 4 Department of Medical Research Pyin Oo Lwin Branch, Pyin Oo Lwin, Myanmar; 5 Department of Convergence Medicine, University of Ulsan College of Medicine and Asan Institute for Life Sciences, Asan Medical Center, Seoul, 05505 Republic of Korea

**Keywords:** Malaria, Microscopic examination, Species-specific nested PCR, Diagnosis, Myanmar

## Abstract

**Background:**

Accurate diagnosis of *Plasmodium* infection is crucial for prompt malaria treatment and surveillance. Microscopic examination has been widely applied as the gold standard for malaria diagnosis in most part of malaria endemic areas, but its diagnostic value has been questioned, particularly in submicroscopic malaria. In this study, the diagnostic performance of microscopic examination and nested polymerase chain reaction (PCR) was evaluated to establish optimal malaria diagnosis method in Myanmar.

**Methods:**

A total of 1125 blood samples collected from residents in the villages and towns located in Naung Cho, Pyin Oo Lwin, Tha Beik Kyin townships and Mandalay of Upper Myanmar were screened by microscopic examination and species-specific nested PCR method.

**Results:**

Among the 1125 blood samples, 261 samples were confirmed to be infected with malaria by microscopic examination. Evaluation of the 1125 samples by species-specific nested PCR analysis revealed that the agreement between microscopic examination and nested PCR was 87.3% (261/299). Nested PCR successfully detected 38 *Plasmodium falciparum* or *Plasmodium vivax* infections, which were missed in microscopic examination. Microscopic examinations also either misdiagnosed the infected *Plasmodium* species, or did not detect mixed infections with different *Plasmodium* species in 31 cases.

**Conclusions:**

The nested PCR method is more reliable than conventional microscopic examination for the diagnosis of malaria infections, and this is particularly true in cases of mixed infections and submicroscopic infections. Given the observed higher sensitivity and specificity of nested PCR, the molecular method holds enormous promise in malaria diagnosis and species differentiation, and can be applied as an effective monitoring tool for malaria surveillance, control and elimination in Myanmar.

## Background

Accurate diagnosis of malaria is important for providing realistic estimates of malaria burden and preventing misinformed interventions [1, 2]. For over a century, microscopic examination of thick and thin blood smears has been considered the gold standard for the diagnosis of malaria in most malaria-endemic regions due to its ability to identify infected *Plasmodium* species and quantify parasitaemia levels at a low cost [3]. However, this method has several limitations in that it is laborious and time-consuming, and requires the availability of well-trained and highly qualified microscopists. Misdiagnosis can occur frequently in cases of low parasitaemia and incorrect species identification, and this can lead to incorrect treatment [4]. To overcome these limitations, several alternative methods for malaria diagnosis have been developed. One of these is a molecular detection method based on the amplification of parasitic DNA [5–9]. Polymerase chain reaction (PCR)-based diagnostic protocols have been recognized as powerful tools to detect mixed *Plasmodium* species infections and differentiate the infected species with high specificity and sensitivity [10–13]. Nevertheless, PCR methods have some limitations, such as high cost and low applicability in rural areas or field-based settings without adequate laboratory equipment [14]. Rapid diagnostic tests (RDTs) for parasite antigen detection provide reliable results in a short time-period and offer a useful alternative to microscopy in conditions where microscopic examination is not feasible [15–17]. Due to these advantages, RDTs have been introduced as a diagnostic tool in many malaria-endemic areas. However, several important issues remain to be addressed, including diagnostic accuracy, high cost, and performance efficacy under unfavorable field conditions. The performance of RDTs can also be affected by the detection of residual parasite antigens from previous infections, leading to a false positive result, and deletions or mutations within the parasite antigen, resulting in a false negative [18, 19]. Moreover, currently available RDTs do not allow quantification and differentiation of *Plasmodium* species other than *Plasmodium falciparum* and *Plasmodium vivax*.

The incidence of malaria in Myanmar has decreased remarkably in recent years, but malaria is still one of the major public health concerns in the country. Myanmar accounts for approximately 77% of the estimated cases and 79% of the estimated malaria deaths in the Greater Mekong Subregion [20]. Moreover, the malaria burden and the emergence of drug resistant malaria parasites along the international border areas of Myanmar are significant challenges for the National Malaria Control Programme [21–24]. All *Plasmodium* species associated with human infection, *P. falciparum*, *P. vivax*, *Plasmodium malariae*, and *Plasmodium ovale*, are considered to occur in Myanmar [25, 26]. *Plasmodium knowlesi* infections have also occurred in some areas, predominantly as a co-infection with either *P. falciparum* or *P. vivax* [27]. The combined use of microscopic examination and RDTs has been widely implemented as a mean to diagnose malaria in endemic areas of Myanmar. However, it is not easy to clearly identify infected *Plasmodium* species and mixed infections with different malaria parasites using these two diagnostic methods. Moreover, information on the diagnostic efficiency of these diagnostic methods is also highly limited.

In this study, the accuracy of routine microscopic examination was analysed by comparing to species-specific nested PCR detection method. PCR method produced conflicting results, particularly in cases of mixed infections with different *Plasmodium* species and submicroscopic infections. These results suggest that molecular diagnostic approach is more reliable than microscopic examination for the accurate diagnosis of *Plasmodium* species as a part of the malaria surveillance programs in Myanmar.

## Methods

### Study areas and microscopic examination

**Fig. 1:**
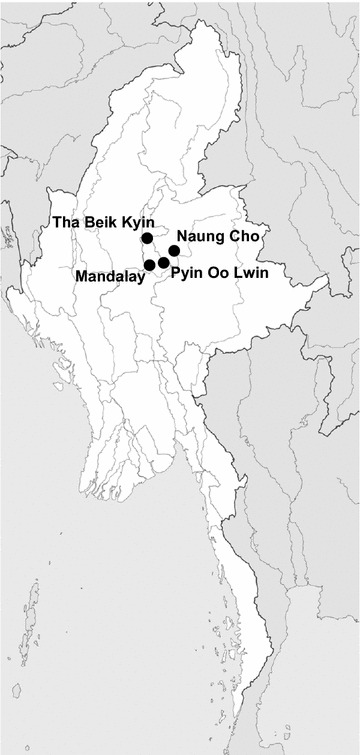
Map of the study sites. Between August 2013 and December 2015, field surveys for malaria were conducted in the regions of Naung Cho, Pyin Oo Lwin, Tha Beik Kyin townships and Mandalay in Upper Myanmar

Between August 2013 and December 2015, field surveys for malaria were conducted in towns and villages located in the regions of Naung Cho, Pyin Oo Lwin, Tha Beik Kyin townships and Mandalay in Upper Myanmar (Fig. 1). These regions were identified as malaria endemics in field surveys over the last few years; malaria transmission was heterogeneous and seasonal with most clinical cases occurred during the rainy season. A total of 1125 residents, 325 suspected malaria patients with clinical symptoms and 800 healthy individuals without any malaria symptom, were enrolled in this study. For the microscopic examination, thick and thin blood smears were prepared from a finger prick, and stained with 10% Giemsa for 15 min. The stained smears were observed with a light microscope at 1000× magnification to confirm the presence of malaria parasites. Microscopic examination of the smears was conducted independently by two experienced microscopists in the Department of Medical Research Pyin Oo Lwin Branch, Myanmar. Agreement between the independent readings of the two microscopists was 99.9% (*p* < 0.001). A slide was considered negative if no parasite was observed in a count of 500 leucocytes.

### Blood collection for molecular analysis

Finger-prick blood samples were taken from 1125 residents. Written consent was obtained from each individual prior to blood collection. Two or three drops of blood (approximately 50 μl) from a finger prick were spotted on Whatman 3 MM filter paper (GE Healthcare, Maidstone, UK) and allowed to air dry for subsequent molecular analysis.

### Ethics

This study was approved by the Ministry of Health, Myanmar, and by the Ethical Review Committee of Inha University School of Medicine, Korea. Informed written consent and permission (in the case of children under 18 years of age) were obtained from each patient or a parent/legal guardian.

### Polymerase chain reaction

Genomic DNA was isolated from each spotted blood filter paper by using the QIAamp DNA Blood Mini Kit (Qiagen, Hilden, Germany). Genomic DNA was eluted with 100 μl of sterile H_2_O. Nested PCR specific to the five human malaria parasite species (*P. falciparum*, *P. vivax*, *P. ovale curtisi*, *P. ovale wallikeri* and *P. malariae*) was performed using a pair of primers targeted to the 18S rRNA gene described previously [5, 6]. *P. knowlesi* was detected by PCR using a set of primers specific to the 18S rRNA gene [28]. For the primary PCR reaction, 2 μl of purified genomic DNA (approximately 10 ng of DNA) were used in a 30 μl reaction. For the nested PCR, 1 μl of the primary PCR reactant were used as a template in a 30 μl reaction. The amplified PCR products were analysed using a 1.5% agarose gel, stained with ethidium bromide, and observed under ultraviolet light. To rule out false positives, the genomic DNA isolated from a healthy Korean individual residing in a region not considered endemic for malaria was included a negative control in all PCR reactions. The presence or absence of different *Plasmodium* species was analysed with species-specific amplicon sizes. Randomly selected PCR products for each *Plasmodium* species were also sequenced to confirm the accuracy of the amplified products.

### Statistic analysis

The 95% confidence intervals (CI) were calculated as appropriate and the value of *P* < 0.05 was considered statistically significant.

## Results

### Microscopic examination

Thin and thick blood smears from a total of 1125 residents including 325 suspected malaria patients were examined for the presence of malaria parasites using microscope. The results of the microscopic examination identified 261 cases (23.2, 95% CI 20.7–25.7) as positive for malaria infection (Table 1). All positive results were obtained in blood samples from 261 suspected malaria patients. The remained 64 suspected malaria patients and 800 non-symptomatic healthy residents were determined to negatives. Of the 261 microscopically confirmed cases, 135 cases (51.7%) were *P. falciparum* mono-infections, 94 cases (36.0%) were *P. vivax* mono-infections, and 31 cases (11.9%) were *P. falciparum* and *P. vivax* mixed infections. There was a case (0.4%) confirmed as a *P. malariae* mono-infection.

**Table 1:** Results for microscopic examination

	Microscopic examination					
	Pf	Pv	Pf/Pv	Pm	Negative	Total
Suspected patients	135	94	31	1	64	325
Healthy individuals	0	0	0	0	800	800
Total	135	94	31	1	864	1125

Pf, *Plasmodium falciparum*; Pv, *Plasmodium vivax*; Pf/Pv, mixed with *P. falciparum* and *P. vivax*; Pm, *Plasmodium malariae*

### Species-specific nested PCR analysis

**Fig. 2:**
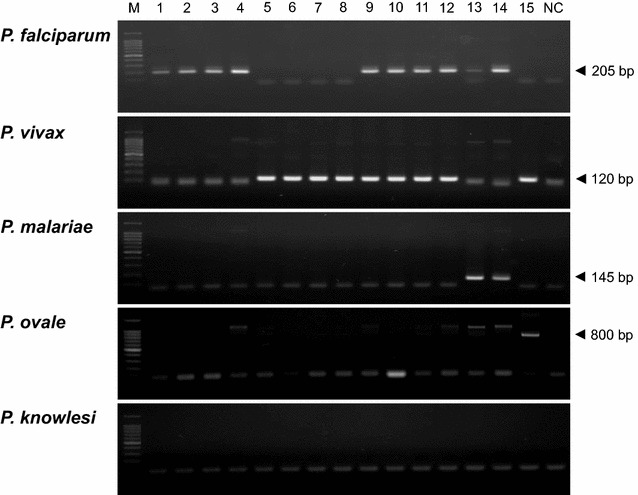
Identification of *Plasmodium* species by species-specific nested PCR. A total of 299 blood samples were confirmed to positive by species-specific nested PCR. Representative results are presented. Lane M, 100-bp ladder;* lanes 1*–*4*, *Plasmodium falciparum* (205 bp amplicon);* lanes 5*–*8*, *Plasmodium vivax* (120 bp amplicon);* lanes 9*–*12*, mixed with *P. falciparum* and *P. vivax*;* lanes 13*–*14*, mixed with *P. falciparum* and *Plasmodium malariae* (145 bp amplicon);* lane 15*, mixed with *P. vivax* and *Plasmodium ovale curtisi* (800 bp amplicon); lane NC, negative control. No positive case was detected for *Plasmodium knowlesi*

The 1125 blood samples were analysed for the presence of *Plasmodium* DNA by species-specific nested PCR. The representative species-specific nested PCR amplification results for the *Plasmodium* species are shown in Fig. 2. Correct amplifications for the sequences from each *Plasmodium* species were confirmed by nucleotide sequencing analysis of randomly selected PCR products. *Plasmodium* infection was confirmed in 299 cases (26.6, 95% CI 24.0–29.2%). The 261 positive cases were corresponded to the samples detected by microscopic examination, but the 38 positive cases were negative in the microscopic examination. The specific *Plasmodium* species were identified as follows: *P. falciparum* (140 cases), *P. vivax* (99 cases), *P. malariae* (1 case), *P. ovale curtisi* (2 cases), and mixed infection with *P. falciparum* and *P. vivax* (52 cases), mixed infection with *P. falciparum* and *P. malariae* (4 cases), and mixed infection with *P. vivax* and *P. ovale curtisi* (1 case) (Table 2). No case of *P. knowlesi* or *P. ovale wallikeri* infection was detected in the analysed samples.

**Table 2:** Results for species-specific nested PCR

	Species-specific PCR								
	Pf	Pv	Pm	Po	Pf/Pv	Pf/Pm	Pv/Po	Negative	Total
Suspected patients	117	84	1	2	52	4	1	64	325
Healthy individuals	23	15	0	0	0	0	0	762	800
Total	140	99	1	2	52	4	1	826	1125

Pf, *Plasmodium falciparum*; Pv, *Plasmodium vivax*; Pm, *Plasmodium malariae*; Po, *Plasmodium ovale*; Pf/Pv, mixed with *P. falciparum* and *P. vivax*; Pf/Pm, mixed with *P. falciparum* and *P. malariae*; Pv/Po, mixed with *P. vivax* and *P. ovale*

### Comparison of microscopic examination and nested PCR for *Plasmodium* detection

The results of the nested PCR analysis were different from those of the microscopic examination, and an overall comparison of the results for these active malaria detection methods is summarized in Table 3. There was 87.3% (261/299) agreement between PCR and microscopy regarding the parasite species identification in the analysed blood samples. However, microscopic examination misdiagnosed the *Plasmodium* species, or did not detect mixed infections in 31 samples. Neither mono-infection nor mixed infections of *P. ovale* were detected by microscopic examination. However, 2 *P. ovale curtisi* mono-infections and a mixed infection of *P. ovale curtisi* and *P. vivax*, which were misdiagnosed to *P. vivax* infections in microscopic examination, were detected by nested PCR. Moreover, 4 cases of *P. falciparum* and *P. malariae* mixed infections were detected by nested PCR analysis, which were misdiagnosed as *P. falciparum* mono-infections or mixed infections with *P. falciparum* and *P. vivax* under microscopy. The number of *P. falciparum* and *P. vivax* mixed infections also increased from 31, based on the microscopic examination, to 52 in the nested PCR analysis. A total of 38 among the 864 microscopy negative samples were also identified to *P. falciparum* (23 cases) or *P. vivax* (15 cases) infections. These results suggest that the molecular approach is more sensitive and reliable than microscopic examination, particularly in the case of mixed infections and submicroscopic infections.

**Table 3:** Summary of PCR results compared with microscopic examinations

Microscopy		PCR							
	N	Pf	Pv	Pm	Po	Pf/Pv	Pf/Pm	Pv/Po	Negative
Pf	135	117	2	0	0	13	3	0	0
Pv	94	0	82	0	2	9	0	1	0
Pm	1	0	0	1	0	0	0	0	0
Pf/Pv	31	0	0	0	0	30	1	0	0
Negative	864	23	15	0	0	0	0	0	826
Total	1125	140	99	1	2	52	4	1	826

Pf, *Plasmodium falciparum*; Pv, *Plasmodium vivax*; Pm, *Plasmodium malariae*; Po, *Plasmodium ovale*; Pf/Pv, mixed with *P. falciparum* and *P. vivax*; Pf/Pm, mixed with *P. falciparum* and *P. malariae*; Pv/Po, mixed with *P. vivax* and *P. ovale*

## Discussion

Accurate diagnosis of the *Plasmodium* species is not only important for establishing a successful treatment regimen, but also for designing effective malaria control measures in endemic regions where multiple species of malaria parasites exist. Misidentification of the *Plasmodium* species could cause a severe public health concerns since it could extend the parasite clearance times and lead to recrudescence and drug resistance [29–31]. Conventional microscopic examination by qualified microscopists has been adopted as the primary method for malaria diagnosis and surveillance in Myanmar. Recently, RDTs were introduced as effective screening tools in field-based malaria surveys since they provide readily available results, and thereby enable rapid treatment. However, only limited information is available regarding the effectiveness of the parasite detection techniques that are widely used in Myanmar. As all common human malaria parasites, as well as *P. knowlesi*, have been shown to exist in Myanmar [25–27, 32], correct diagnosis of the infected *Plasmodium* species and prompt treatment are essential to malaria control and elimination in the country. Therefore, studies are needed to assess quality and impact of these diagnostic methods for optimal malaria diagnosis setting and nationwide malaria surveillance outcomes.

In this study, 1125 blood samples collected from residents in malaria endemic areas in Upper Myanmar were analysed using microscopic examination and species-specific nested PCR. Overall malaria incidence as determined by microscopic examination and nested PCR method were relatively similar, with a coincidence of 87.3%, but important discrepancies between the microscopic examination and nested PCR analysis were also identified. The role of microscopic examination as the gold standard for malaria diagnosis has been questioned due to false negative results at low parasitaemiae levels (less than 20–30 parasites/μl of blood) and frequent errors in species identification in mixed infections [33]. The low diagnosis reliability of microscopy for species-specific and mixed-infections in endemic areas has been reported previously [12, 34–39]. Consistent with these previous reports, mixed infections that were missed in the microscopic examination were identified in 27 samples by molecular detection method. In particular, the prevalence of *P. falciparum* and *P. vivax* mixed infections highly increased with nested PCR analysis. Misdiagnosis using microscopic examination technique can easily occur, particularly in the case of mixed infections where one parasite species is dominant over the others [40]. It has been postulated that co-existence of *P. falciparum* and *P. vivax* in a single human host results in parasitic suppression through interspecies inhibition [41–43]. The clinical symptoms in mixed infections can also vary depending on whether it is a *P. falciparum* or *P. vivax* super-infection. Although it still remains controversial whether mixed infections involving *P. falciparum* or *P. vivax* and other *Plasmodium* species are beneficial or harmful [44, 45], acute diagnosis of malaria parasites in mixed infections followed by prompt treatment is essential to the prevention of relapses or recrudescences, and the associated severe complications.

Asymptomatic infection, particularly in the cases of *P. falciparum* and *P. vivax*, has been recognized an important obstacle to controlling malaria since asymptomatic carriers do not seek treatment for the infection, so that they constitute a reservoir available for new infections. A significant proportion of *P. falciparum* and *P. vivax* infections are asymptomatic or subclinical in malaria endemic countries [46–52]. Repeated exposure and acquisition of partial immunity can result in persistent low-grade infection, even though infected individuals may not have typical malaria symptoms such as fever and anemia. Consistent with these previous reports, 38 asymptomatic cases (22 cases of *P. falciparum* and 15 cases of *P. vivax*), which were not detected by microscopic examination, were identified by molecular detection method. These results suggest that the importance of detection of asymptomatic infections with submicroscopic level in Myanmar for successful malaria control and elimination in the country. Aggressive approach to detect asymptomatic carriers followed by prompt treatment would be required.

The results of this study also provide partial epidemiological information regarding the prevalence of *Plasmodium* species in the regions studied. Based on the results from the nested PCR analysis, overall malaria infection rate in the residents in the studied endemic areas was 26.6% (299/1125). It has been estimated that total malaria cases confirmed either by microscopy examination or by RDT in Myanmar were 420,808 in 2010 [53]. Most of the confirmed cases were *P. falciparum* and *P. vivax* infections (420,462 cases) and the ratio of *P. falciparum*/*P. vivax* infections was 70% [53]. Consistent with the previous results, *P. falciparum* and *P. vivax* accounted for most of the PCR-confirmed cases in this study. But the ratio of *P. falciparum*/*P. vivax* was not as high as previously reported and a high proportion of mixed *P. falciparum* and *P. vivax* infections (17.4%) were identified. It is assumed that *P. vivax* is becoming predominant species in the studied areas with the recent decrease of *falciparum* malaria cases. Asymptomatic and submicroscopic infections by the two *Plasmodium* species were also a great concern. The prevalence of *P. malariae* and *P. ovale* was low, but single as well as mixed infections involving either *P. falciparum* or *P. vivax* were identified. Distribution of *P. malariae* and *P. ovale* in Myanmar has been reported previously [25, 26, 54]. In particular, 8 confirmed *P. ovale* cases (mixed infections with *P. vivax* and *P. malariae*) were reported in Pyin Oo Lwin area [25]. These observations, along with the present study, suggest that *P. malariae* and *P. ovale* are endemic in Upper Myanmar, though the prevalence is not high. *P. malariae* and *P. ovale* infections can be easily missed to diagnose by microscopic examination, primarily due to quite low parasitaemia of the parasites [55, 56]. Moreover, in mixed infections with other *Plasmodium* species, it is more difficult to differentiate the parasites from a background with large numbers of parasites of other species [55, 56]. In fact, the *P. malariae* and *P. ovale* infections that were not detected or misread in microscopic examination were successfully identified by nested PCR methods in this study. High rates of *P. knowlesi* co-infection with other malaria parasites also has been reported in North-Eastern Myanmar [27], but no cases of *P. knowlesi* mono- or co-infection were detected in this study. It is worthy to realize the limitation of PCR-based molecular diagnosis methods to detect parasite DNA, especially in very low level of parasitaemia. A routine extraction process of parasite DNA from dried blood filters could result in loss or dilution of parasite DNA for successful amplification [36]. Nevertheless, as confirmed in this study and other previous studies [12, 34–39], PCR-based methods showed greater diagnostic performance than conventional microscopic examination to detect *Plasmodium* species with high specificity and sensitivity. Molecular diagnostic approach is highly recommended for the accurate diagnosis of *Plasmodium* species as a part of the malaria surveillance and elimination programs in Myanmar.

## Conclusions

This study has shown that microscopic examination does not reliably distinguish *Plasmodium* species in the studied areas co-endemic for multiple *Plasmodium* species. Nested PCR not only detected all microscopy-positive samples, but also detected single or mixed infections and submicroscopic infections that were missed or misread by microscopy. Given the observed higher sensitivity and specificity associated with the nested PCR technique, molecular detection method holds enormous promise for malaria diagnosis and can be used as effective monitoring tools for malaria surveillance, control and elimination in Myanmar. Submicroscopic and asymptomatic infections also highlight the urgent need to develop new public health strategies and appropriate community-based interventions for the effective control and elimination of malaria in Myanmar.
